# Pneumococcal meningitis and COVID-19: dangerous coexistence. A case report

**DOI:** 10.1186/s12879-022-07156-1

**Published:** 2022-02-23

**Authors:** Katarzyna Guziejko, Piotr Czupryna, Ewa Katarzyna Zielenkiewicz-Madejska, Anna Moniuszko-Malinowska

**Affiliations:** 1 2nd Department of Lung Diseases and Tuberculosis, Medical University of Bialystok, Żurawia 14, 15-540 Białystok, Poland; 2 Department of Infectious Diseases and Neuroinfections, Medical University of Białystok, Żurawia 14, 15-540 Białystok, Poland; 3 Observation and Infectious Department, Independent Public Healthcare Center in Bielsk Podlaski, ul. Kleszczelowska 1, 17-100 Bielsk Podlaski, Poland

**Keywords:** COVID-19, *Streptococcus pneumoniae*, Meningitis, Sepsis, Co-infection

## Abstract

**Background:**

SARS-CoV-2 is the major cause of infections in humans since December 2019 and is top of the global health concern currently. *Streptococcus pneumoniae* is one of the leading pathogens of invasive bacterial diseases, including pneumonia, sepsis, and meningitis. Moreover, this bacteria is mostly responsible for secondary infections subsequent to post-viral respiratory disease. Co-infections with bacterial and viral pathogens are associated with severe course of the disease and are a major cause of mortality. In this report, we describe a rare case of COVID-19 patient with pneumococcal sepsis and meningitis of unsuccessful course.

**Case presentation:**

A 89-year-old man, not vaccinated against SARS-CoV-2 infection, was diagnosed with COVID-19 pneumonia. Patient required oxygen therapy due to respiratory failure. The initial treatment of viral infection with tocilizumab and dexamethasone allowed for the stabilization of the patient’s condition and improvement of laboratory parameters. On the 9th day of hospitalization the patient’s condition deteriorated. Consciousness disorders and acute respiratory disorders requiring intubation and mechanical ventilation were observed. Brain computed tomography excluded intracranial bleeding. The *Streptococcus pneumoniae* sepsis with concomitant pneumoniae and meningitis was diagnosed based on microbiological culture of blood, bronchial wash, and cerebrospinal fluid examination. Despite targeted antibiotic therapy with ceftriaxone and multidisciplinary treatment, symptoms of multiple organ failure increased. On the 13th day of hospitalization, the patient died.

**Conclusions:**

Co-infections with bacterial pathogens appear to be not common among COVID-19 patients, but may cause a sudden deterioration of the general condition. Not only vascular neurological complications, but also meningitis should be always considered in patients with sudden disturbances of consciousness. Anti-inflammatory treatment with the combination of corticosteroids and tocilizumab (or tocilizumab alone) pose a severe risk for secondary lethal bacterial or fungal infections. Thus, treating a high-risk population (i.e. elderly and old patients) with these anti-inflammatory agents, require daily clinical assessment, regular monitoring of C-reactive protein and procalcitonin, as well as standard culture of blood, urine and sputum in order to detect concomitant infections, as rapidly as possible.

## Background

From December 2019, coronavirus disease-2019 (COVID-19) caused by a newly discovered coronavirus (severe acute respiratory syndrome coronavirus-2, SARS-CoV-2), is not only the most common infectious disease, but also the major concern of global health [1, 2]. *Streptococcus pneumoniae* (*S. pneumoniae*), along with *Haemophilus influenzae* (*H. influenzae*) and *Neisseria meningitidis* (*N. meningitidis*), are the main pathogens of most cases of invasive bacterial diseases, including pneumonia, sepsis and meningitis [3, 4]. Based on the studies conducted so far during influenza virus pandemic, *S. pneumoniae* has been proven to be mostly responsible for secondary infections subsequent to post-viral respiratory disease [5–7]. The incidence of co-infections with bacterial and viral pathogens are estimated at 3–8.6%. However, their clinical course is associated with severe symptoms and is the major cause of mortality [8–10].

In this report, we present a rare case of patient with COVID-19, pneumococcal sepsis and meningitis of unsuccessful course. No symptoms of invasive bacterial co-infection were found during admission of patient to the hospital. Deterioration of the general condition and the occurrence of disturbances of consciousness required a differential diagnosis between the neurological complications of COVID-19 and neuroinfection. Despite early diagnosis and appropriate treatment, the course of viral and bacterial co-infection was fatal in the described case.

## Case presentation

A 89-year-old male patient, farmer, not vaccinated against COVID-19, was admitted to the Observation and Infectious Diseases Department District Hospital due to fever, cough, weakness, and low exercise tolerance. Symptoms exacerbated during last 14 days prior to hospitalization. He was treated empirically with oral antibiotics in standard doses (amoxicillin and clavulanic acid, levofloxacin) without clinical improvement. Rapid test for qualitative detection of SARS-CoV-2 antigen (Panbio™ COVID-19 Ag Rapid Test Device, Abbott), performed from nasopharyngeal swabs during admission in the Emergency Room, was positive. In medical history patient reported bronchial asthma, hypertension, and heart failure. No neurodegenerative condition or dementia have been diagnosed previously.

**Fig. 1:**
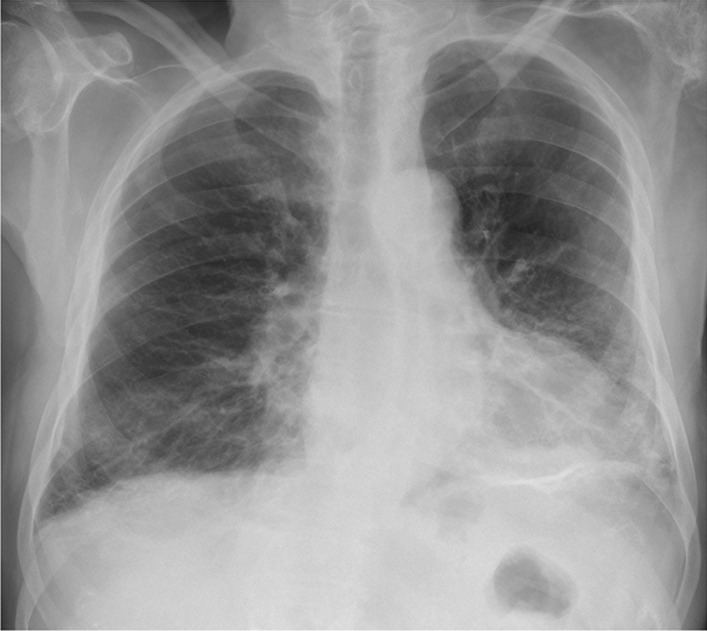
Chest x-ray: bilateral, diffused interstitial opacities in lower lobes, especially by the left side

**Fig. 2:**
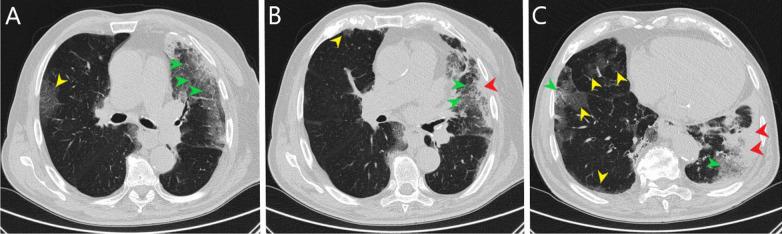
Chest high resolution computed tomography (**A**–**C** lung window, axial scans): bilateral, predominately peripheral, ground-glass opacities (yellow arrowheads), crazy paving (green arrowheads) and consolidations (red arrowheads) especially in left lower lobe

During admission, the patient was conscious and in logic contact. Physical examination revealed dehydration, prolonged exhalation phase, numerous crackles and single wheezes during lung’s auscultation, finger pulse SpO2 was 85% without oxygen supplementation. Laboratory tests showed increased concentration of C-reactive protein (CRP) with normal procalcitonin (PCT) values, high interleukin 6 (IL-6) and lactic acid value, increased activity of transaminases (Table 1). Severe hypoxemia was found in blood gas analysis. The chest X-ray revealed bilateral, diffused interstitial opacities, especially in the left lower lobe (Fig. 1). High resolution computed tomography (HRCT) of chest confirmed typical for COVID-19 pneumonia radiological findings. In total, right lung was affected in 40%, and the left in 60% (Fig. 2). The treatment included tocilizumab (single dose of 600 mg), low molecular weight heparin (enoxaparin 1 mg per kg bodyweight daily), glucocorticosteroids (dexamethasone 6 mg per day), bronchodilators (SABA + SAMA), fluid therapy, passive oxygen therapy via a mask with an oxygen reservoir with a flow of 6 L/min. Stabilization of the patient's condition, normalization of blood saturation and inflammatory parameters in laboratory tests were achieved (Table 1). Fever was not observed.

**Table 1:** Results of laboratory tests

Day of hospitalization	1	7	9	9	10	11	12
CRP [mg/l] Normal range < 0.5 mg/l	76.23	4.09	53.26	62.77	107.99	91.56	51.15
PCT [ng/ml] Normal range < 0.5 mg/l	0.16	–	0.85	2.61	8.7	13.07	8.37
WBC count [cells/μl] Normal range 4.0–10.0 × 10^3^/μl	7.3	13.47	26.0	22.98	22.99	21.25	16.30
Lymph. [cells/μl] Normal range 0.9–4.5 × 10^3^/μl	0.3	0.22	0.78	0.19	1.15	0.36	14.79
Neutr. [cells/μl] Normal range 1.8–7.7 × 10^3^/μl	5.7	12.45	24.18	21.9	20.69	19.59	0.32
RBC count [cells/μl] Normal range 4.63–6.08 × 10^6^/μl	5.68	5.0	5.55	5.15	5.03	4.66	4.7
HGB [g/dl] Normal range 14.00–18.00 g/dl	16.2	14.5	16.4	15.0	14.6	13.7	13.6
PLT count [cells × 103/μl] Normal range 150–400 × 10^3^/μl	218	245	258	233	161	150	129
AlAT [IU/l] Normal range < 41 IU/l	54	45	32	24	35	38	39
AspAT [IU/l] Normal range < 35 IU/l	57	26	24	19	69	57	41
GLUC [mmol/l] Normal range 4.6–6.4 mmol/l	8.5	7.0	11.0	–	10.1	–	15.3
Lactic acid [mmol/l] Normal range 0.5–2.2 mmol/l	3.4	–	3.0	–	3.2	–	4.2
CREA [ml/ml] Normal range 0.7–1.2 mg/l	1.19	0.79	1.12	1.55	3.23	4.52	5.00
D-dimer [μg/ml] Normal range < 0.5 μg/ml	2.09	1.89	4.35	15.69	37.36	7.51	3.76
IL-6 [pg/ml] Normal range < 7.0 pg/ml	119.2	–	–	–	–	–	–

*CRP* C-reactive protein, *PCT* procalcitonin, *WBC* white blood cell count, *Lymph.* lymphocyte count, *Neutr.* neutrophil count, *RBC* red blood cell count, *HGB* hemoglobin, *PLT* platelets count, *AlAt* alanine aminotransferase, *AspAt* aspartate aminotransferase, *GLUC* glucose concentration, *CREA* creatinine, *D-dimer* fibrin degradation product, *IL-6* interleukin 6

**Fig. 3:**
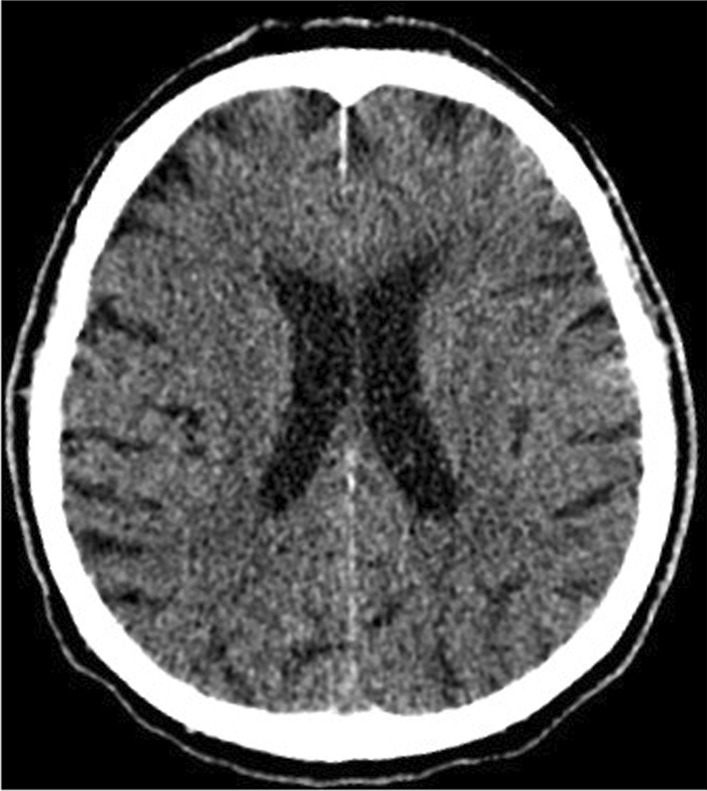
Brain computed tomography (axial scan): no evidence of intracranial hemorrhage. Features of leukoaraiosis. Ventricles of normal volume. Brain structures without displacement. Skull bones in the study area without traumatic injuries

On the 9th day of hospitalization, in the early morning hours, the patient's condition deteriorated. He was unconscious, with no reaction to external stimuli, rated at 5 points on the Glasgow scale. The physical examination revealed fever up to 38.2 °C, tachypnea (respiratory rate was 30 breaths per minute), high blood pressure values, right hemiparesis, the neck stiffness, a positive Babinski sign on the right sight. Due to the suspicion of a stroke, resulting from thromboembolic complications of COVID-19, a brain CT was performed. No signs of intracranial bleeding were observed (Fig. 3). Laboratory tests revealed a significant increase in inflammatory parameters (Table 1). Blood and urine samples were collected for microbiological tests. Empirical antibiotic therapy with ceftriaxone (2 g twice a day intravenously) and ciprofloxacin (400 mg twice a day intravenously) was implemented. Six hours after the onset of consciousness disturbances, the patient developed apnea. He was intubated and mechanical ventilation was started. Control brain CT performed 12 h after first brain imagining excluded intracranial hemorrhage. Blood and the bronchial wash cultures were positive for *S. pneumoaniae* after 48 h. Minimum inhibitory concentration (MIC) for ceftriaxone was ≤ 0.12 μg/mL. Urine culture was negative. Lumbar puncture was performed after stabilization of the patient's condition. Based on the result of the cerebrospinal fluid (CSF) tests, bacterial neuroinfection was confirmed (Table 2). CSF culture was negative. No PCR and rapid latex agglutination tests of CSF were performed because of the small amount of the collected fluid. Despite the multidisciplinary treatment, the patient's condition deteriorated. Symptoms of multiple organ failure increased. On the 13th day of hospitalization, the patient died.

**Table 2:** Results of CSF examination

Parameter	Result	Normal range
Pleocytosis (cells/μl)	2420	0–5
Lymphocytes (%)	5	
Granulocytes (%)	86	
Monocytes (%)	6	
Macrophages (%)	3	
Protein concentration (mg/dl)	428.9	15.00–45.00
Glucose concentration (mmol/l)	4.71	2.22–3.89
CSF culture	no growth	

## Discussion and conclusions

COVID-19 pneumoniae and the respiratory failure are the most common clinical manifestations among hospitalized patients with SARS-CoV-2 infection. Complications of COVID-19 include ARDS, sepsis, septic shock, coagulopathy, acute cardiac injury, renal failure [1, 2].

The increasing prevalence of neurological manifestations and complications associated with COVID-19 have been also observed. Current literature suggests that gustatory and olfactory dysfunctions, myalgia, headache, altered mental status, confusion, delirium, dizziness, nausea and vomiting, as well as stroke, cerebral venous thrombosis, seizures, meningoencephalitis, Guillain–Barré syndrome are most common neurological symptoms and manifestations in COVID-19 patients [11]. They can occur prior, during, and even after acute phase of SARS-CoV-2 infection [12].

However, in the presence of life-threatening respiratory failure in critically ill COVID-19 patients, neurological manifestations are usually undiagnosed [13]. In our case, the cause of sudden worsening of the neurological symptoms and the deterioration of the patient's condition was meningitis. Microbiological cultures of various samples and CSF examination resulted in early confirmation of neuroinfection. Simultaneous imaging of the brain excluded vascular complications, which may also appear  in the course of SARS-CoV-2 infections [12].

*Streptococcus pneumoniae* is one of leading pathogens of invasive bacterial diseases, including pneumonia, sepsis, and meningitis. [3, 4]. This pathogen is responsible for 25.1–41.2% of meningitis cases among all age groups. This clinical manifestation of bacterial infection is burdened with high mortality. Early diagnosis and the initiation of therapy significantly affect the course of the disease. Untreated properly, can be fatal in most cases [4].

Brueggemann et al. compared the incidence of invasive bacterial infection with *S. pneumoniae*, *H. influenzae*, and *N. meningitidis* during the COVID-19 pandemic in 26 countries with rates in previous years. They confirmed a significant reduction in invasive diseases in early 2020. The incidence of reported *S. pneumoniae* infections decreased by 68% at 4 weeks since the pandemic restrictions were introduced for global scale [14]. Despite the decrease in the incidence of bacterial invasive infection, the suspicion of meningitis should be taken into account in the differential diagnosis of a disturbed consciousness, accompanied by acute respiratory failure, fever and a significant increase of inflammatory parameters. The clinical course of the neuroinfection was so severe in our patient, that mechanical support of ventilation was necessary to stabilize the patient’s condition. Only then, after exclusion of cerebral edema in CT scans, it was possible to perform a lumbar puncture to confirm meningitis.

Coronaviruses are known for their neurological tropism. Numerous reports based on previous SARS-CoV and MERS-CoV epidemics, provide clear evidence of various neurologic sequalae (i.e. encephalitis, seizures, encephalopathy, Guillain–Barre syndrome), which may occur in association with respiratory symptoms [15, 16]. Neuroinvasion may take the form of viral encephalitis, confirmed by the presence of viral RNA in the CSF in single cases [17, 18]. CSF examinations in COVID-19-associated meningoencephalitis show pleocytosis with predominance of lymphocytes and increased concentration of protein, which typical for viral infections. This suggests active intrathecal inflammation [18]. In our patient result of CSF examination indicated bacterial etiology of neuroinfection.

Although classic abnormalities of CSF examination typically for bacterial meningitis was observed and *S. pneumoniae* was isolated from blood and bronchial wash, CSF culture was negative. Similar observations have been previously reported in literature [19]. Bohr et al. in the retrospective study in 875 patients diagnosed with bacterial meningitis, not pretreated with antibiotics, showed positive CSF culture in 85% cases [20]. In described case, a negative CSF culture could have been the result of using antibiotics before the lumbar puncture. The results of two large cohort studies confirmed a 4–18% decrease in culture positivity when empiric antibiotic therapy was administered prior to CSF sampling [20, 21].

Based on the studies conducted so far, it has been proven that viruses may promote the development of severe invasive bacterial infections, including meningitis, in selected patients [5, 6].

Klein et al. study concluded that bacterial co-infection should be considered in differential diagnosis in all patients hospitalized with influenza, but not all patients are co-infected. The predominant co-infecting organism was *S. pneumoniae* followed by *Staphylococcus aureus*. Bacterial co-infection is associated with more severe symptoms and higher mortality [7].

Identified bacterial or fungal co-infections among COVID-19 patients are not so common. Langford et al. meta-analysis estimated the presence of co-infections in 8.6% of over 30,000 analyzed patients [8]. Also Palanisamy et al. reported secondary bloodstream infections in 8.5% COVID-19 ICU patients [22]. Similar findings was proved in Rawson’s study of eighteen full texts reporting bacterial/fungal co-infection. Furthermore, they highlighted wide use of broad-spectrum antibiotics, despite the lack of microbiological confirmation of bacterial co-infection [9]. Low rates of bacterial and fungal infection in COVID-19 patients were also reported from Spain and United Kingdom. Bacterial and fungal co-infections and superinfections in these countries were estimated at 3% and 6%, respectively [10].

Tocilizumab increases the risk of opportunistic and serious bacterial infection [23]. In patients 65 years of age or older, receiving doses of 8 mg/kg, the incidence rates of infections reach 8.5 episodes per 100 patient-years. Other tocilizumab-related infection risk factors are coexisting lung disease and corticosteroid therapy [24]. Therefore, regular clinical assessment of patients treated with anti-IL-6 agents is essential for the early diagnosis of developing infection [23].

In presented case, temporary improvement in general condition was achieved after implementation of targeted on cytokine storm treatment (tocilizumab plus dexamethasone), typical for the late phase of COVID-19. Pneumococcal pneumoniae, sepsis and meningitis were diagnosed during hospitalization, although no symptoms of invasive infection were found upon admission to the hospital. The course of co-infection with the SARS-CoV-2 and *S. pneumoniae* was life-threatening. The implemented antibiotic therapy, despite *S. pneumoniae * sensitivity to ceftriaxone (MIC ≤ 0.12 μg/ml), did not result in recovery. The patient died on the 5th day after the onset of pneumococcal infection.

Based on our experience we concluded, that co-infections with bacterial pathogens appear to be not common among COVID-19 patients, but may cause a sudden deterioration of the general condition. Not only vascular neurological complications, but also meningitis should be always considered in patients with sudden disturbances of consciousness. Anti-inflammatory treatment with the combination of corticosteroids and tocilizumab (or tocilizumab alone) pose a severe risk for secondary lethal bacterial or fungal infections. Thus, treating a high-risk population (i.e. elderly) with these anti-inflammatory agents, require daily clinical assessment, regular monitoring of CRP and procalcitonin, as well as standard culture of blood, urine and sputum in order to detect concomitant infections, as rapidly as possible.

## Data Availability

The raw data can be requested from Corresponding Author: kguziejko@wp.pl.

## Abbreviations

COVID-19: Coronavirus disease-2019
CRP: C-reactive protein
CSF: Cerebrospinal fluid
CT: Computed tomography
ICU: Intensive care unit
IL-6: Interleukin 6
MERS-CoV: Middle East respiratory syndrome coronavirus
MIC: Minimum inhibitory concentration
PCR: Polymerase chain reaction
PCT: Procalcitonin
SABA: Short acting beta agonists
SAMA: Short acting muscarinic antagonist
SARS-CoV-2: Severe acute respiratory syndrome coronavirus-2
